# Types and Frequencies of Physical Activity Associated with Physical Fitness in Chinese Children and Adolescents: A National Cross-Sectional Study

**DOI:** 10.3390/healthcare13192400

**Published:** 2025-09-24

**Authors:** Linjie Wei, Zhe Li, Yuliang Sun

**Affiliations:** 1 School of Physical Education, Xi’an University of Architecture and Technology, Xi’an 710055, China; weilinjie@xauat.edu.cn; 2 College of Information and Control Engineering, Xi’an University of Architecture and Technology, Xi’an 710055, China; lizhe@xauat.edu.cn; 3 School of Physical Education, Shaanxi Normal University, Xi’an 710119, China

**Keywords:** physical activity, sedentary behavior, physical fitness, children, adolescents

## Abstract

**Background:** Rising sedentary behavior and declining physical activity (PA) among youth are major public health concerns. Evidence on how different PA types affect fitness in Chinese children is limited. PA indicators were classified according to the Global Matrix 4.0, an international framework for benchmarking PA in children and adolescents. **Objective:** To identify which PA types and frequencies, based on the Global Matrix 4.0, are most associated with physical fitness in Chinese youth. **Methods:** Data from 102,942 children aged 9–18 were analyzed. Descriptive statistics summarized demographics, PA levels, and physical fitness rates. Chi-square tests assessed group differences. Binary logistic regression examined associations between PA and physical fitness, adjusting for gender, age, school level, parental education, and urban–rural residence, with cluster-robust standard errors at the school level. **Results:** Higher frequencies of moderate-to-vigorous physical activity (MVPA), muscle-strengthening exercise (MSE), and organized sports and physical activity (OSPA) were significantly associated with better fitness (*p* < 0.001). OSPA (OR = 1.23) and MSE (OR = 1.21) showed the strongest associations. Sedentary behavior was negatively associated with fitness (*p* < 0.001). **Conclusions:** Regular engagement in MVPA, MSE, and OSPA, alongside reduced sedentary time, promotes physical fitness among Chinese children and adolescents. Policies should support diverse and consistent PA participation.

## 1. Introduction

The global decline in adolescent physical fitness has become a significant public health challenge [1]. According to the World Health Organization, children and adolescents are engaging in less physical activity (PA), while sedentary behaviors are on the rise, contributing to increased risks of obesity, cardiovascular diseases, and mental health problems [2,3,4,5]. As such, finding effective ways to enhance adolescent physical fitness is a pressing issue in global public health [6,7]. Most countries have reported on the overall status of adolescent PA. Previous studies have predominantly focused on the total duration or intensity of PA and its association with physical fitness. At the same time, limited attention has been paid to the differential effects of distinct PA types. This gap makes it unclear which specific forms of PA are most beneficial for improving different components of fitness [8,9,10]. The Global Matrix 4.0, a harmonized international report card on PA for children and youth, categorizes behavioral indicators into five aspects: overall PA, organized sports and physical activity (OSPA), active play, active transportation, and sedentary behavior. Data from 57 countries across six continents are graded from A to F using national surveys or registries (China was one of them), allowing cross-country comparisons and highlighting areas of concern. Incorporating these global benchmarks, this study aims to evaluate the levels of different types of PA—including moderate-to-vigorous physical activity (MVPA), muscle-strengthening exercise (MSE), OSPA, outdoor physical activity (OPA), active transportation and sedentary behavior—and their associations with physical fitness among Chinese children and adolescents aged 9–18, providing context for understanding how China compares internationally and identifying areas for targeted intervention to improve youth health outcomes [11]. Among these activity types, MVPA and MSE are essential for cardiovascular and musculoskeletal health. MSE includes bodyweight and light resistance exercises. Organized sport and physical activity (OSPA) refers to structured and often competitive activities like PE classes or team sports. Active play involves spontaneous, unstructured movement in a playful context. Active transportation includes walking and cycling, supported by policies such as requiring students to live near school.

Despite the support provided by national policies aimed at promoting PA among adolescents, many challenges remain. Cultural differences, academic pressure, urbanization, and varying socioeconomic backgrounds significantly affect adolescents’ PA patterns [12]. Increasing academic pressure has reduced time for extracurricular sports activities and sedentary behavior [13,14]. Lifestyle changes associated with urbanization have further restricted opportunities for outdoor activities, with a growing reliance on motorized transportation instead of active modes such as walking or cycling, all of which may contribute to declines in adolescent physical fitness [15]. Numerous studies have shown that regular MVPA improves children’s and adolescents’ physical fitness. However, there is still a lack of research examining whether different types of PA (e.g., OSPA, active play, active transportation) influence cardiorespiratory endurance, muscle strength, flexibility, and body composition. Addressing this gap, the present study analyses various types and frequencies of PA from the Global Matrix perspective, aiming to explore how different types and frequencies of PA affect the physical fitness of Chinese children and adolescents. This research will provide a solid theoretical foundation for developing more scientifically sound PA promotion strategies to improve global adolescent physical fitness levels.

## 2. Materials and Methods

### 2.1. Sample Information

**Figure 1:**
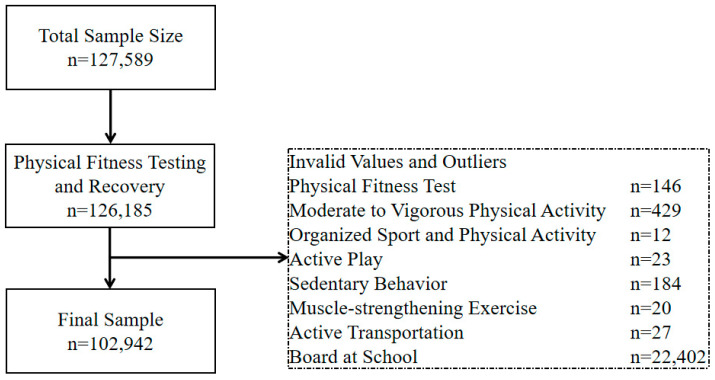
Flowchart of participant inclusion and exclusion in the study.

This is a nationwide cross-sectional study. The participants in this study were selected from the 2019 National Student Physical Fitness and Health Survey and the National Student Physical Fitness Test Cohort, organized by the Ministry of Education of the People’s Republic of China. The survey included 127,589 students from the fourth to twelfth grades (aged 9 to 18 years). After excluding invalid and missing data (Figure 1), 102,942 participants were ultimately included in the analysis. Specifically, 1404 students were excluded due to absence, medical exemption, or incomplete physical fitness testing. An additional 22,943 students were excluded due to missing or invalid responses across key variables, including MVPA (n = 429), OSPA (n = 12), active play (n = 23), sedentary behavior (n = 184), MSE (n = 20), and active transportation (n = 27). Furthermore, 22,402 students who boarded at school were excluded because their structured daily schedules differ substantially from non-boarding students, introducing systemic behavioral variance that may bias associations. This exclusion ensures analytical consistency. The participant selection process was as follows: 30 provinces, municipalities, and autonomous regions of mainland China were included (except Hubei Province due to the COVID-19 pandemic). Cities within each province were divided into four economic tiers based on GDP [16]. One city from each tier was randomly selected as a sampling site, with municipalities directly under the central government selecting one urban district and one suburban district (or county) from each tier. In each chosen site (except for municipalities), two counties (or cities, districts, or banners) were randomly selected as sampling points. From each sampling point, two elementary schools (one urban and one rural), one middle school, and one high school were chosen to comprise the sampled schools. The study was approved by the Academic Committee of Shaanxi Normal University (201916001) on 20 September 2019.

### 2.2. Survey on Participation in Different Types of Physical Activities

Global Matrix 4.0 categorizes PA into three main areas [11]. The Global Matrix 4.0, developed by the Active Healthy Kids Global Alliance (AHKGA), provides an international benchmark for evaluating youth OA, including MVPA, MSE, OSPA, active transportation, outdoor play, and sedentary behavior. Previous studies in China have applied the Global Matrix 4.0 framework to assess PA levels among Chinese children and adolescents, supporting its appropriateness for use in the Chinese population [9,10,17]: (1) Behavioral Indicators (including overall PA, OSPA, active play, active transportation, and sedentary behavior, with an average rating for these indicators); (2) Sources of Influence Indicators (such as the average scores for family and peers, school, community and environment, and government); and (3) Overall Composite Score (the average rating of 10 common indicators). This study focuses on behavioral indicators for several reasons. First, data on behavioral indicators are easier to collect through self-reports or device monitoring, making it a more feasible option given the research resources and time limitations. Second, the research prioritizes children’s and adolescents’ PA behaviors, which are directly linked to health outcomes. Lastly, collecting data on sources of influence indicators (such as policy and environment) is more complex. It may lead to inconsistencies or accuracy issues, while behavioral indicators provide more reliable data, facilitating analysis and interpretation. The questionnaires were completed under the supervision of the assessors, with elementary school students filling them out with the help of teachers. The specific content of the questionnaire is as follows.

#### 2.2.1. Days of MVPA

According to the World Health Organization’s guidelines on PA and sedentary behavior, children and adolescents aged 5 to 17 should accumulate at least 60 min of MVPA daily. Therefore, this study investigated the number of days each individual met the 60 min recommendation for MVPA [18,19]. The question asked was “In the past seven days, how many days did you engage in at least 60 min of MVPA include brisk walking, dancing, etc., where you feel somewhat out of breath, sweaty, or a little tired; Vigorous-intensity activities include fast running, cycling at a rapid pace, or lifting heavy objects, where more effort is required, making you feel out of breath, sweaty, or very tired.)?”. Options were 0 days, 1–2 days,3–4 days, 5–6 days, and 7 days.

#### 2.2.2. Days of Muscle-Strengthening Exercises

This study investigated the number of days each individual engaged in MSE, including bodyweight exercises such as squats, sit-ups, push-ups, and pull-ups, and exercises using equipment like dumbbells, resistance bands, and fitness machines [20]. Although MSEs are not explicitly listed as standalone indicators in Global Matrix 4.0, they are often considered a component of overall PA and are supported by WHO guidelines due to their distinct physiological benefits. Given their growing relevance in adolescent health promotion, MSE was included as a supplementary behavioral indicator in this study to provide a more comprehensive understanding of PA’s relationship with physical fitness. To ensure that the MSE questions were appropriate and understandable for children and adolescents aged 9–18 years, trained researchers administered the questionnaire in classrooms and explained each question in detail. The questions focused on age-appropriate exercises, avoiding heavy weights unsuitable for children, and included options such as bodyweight exercises (squats, push-ups, sit-ups, pull-ups) and light equipment (resistance bands, small dumbbells). MSE is recommended for children and adolescents on at least 3 days per week. The World Health Organization does not specify an exact daily duration for MSE, but regular participation is encouraged to promote musculoskeletal health and overall physical fitness. Participants were asked, “In the past 7 days, how many days did you engage in muscle-strengthening exercises? With response options of 0 days, 1 day, 2 days, 3 days, and ≥5 days.”

#### 2.2.3. Days of OSPA

This refers to the number of days per week participants engaged in organized sports activities, such as physical education classes, sports training, or club activities. The question asked was “In the past 7 days, how many days did you participate in organized physical activities (including physical education classes, after-school sports services, sports training, sports clubs, etc.)?” Options were 0 days, 1–2 days, 3–4 days, 5–6 days, and 7 days.

#### 2.2.4. Active Play (Outdoor PA)

In the Global Matrix, self-directed exercise is divided into two dimensions: the percentage of children and adolescents engaging in unstructured/unorganized active play for more than 2 h per day at any intensity or those spending more than 2 h outdoors. This study selected the second dimension, investigating the number of days individuals spent more than 2 h outdoors. The question asked was “In the past 7 days, how many days did you engage in physical activity for more than two hours?” Options were 0 days, 1–2 days, 3–4 days, 5–6 days, and >7 days.

#### 2.2.5. Active Transportation

This refers to the percentage of children and adolescents who use active transportation methods (such as walking and biking) to travel to and from places like schools, parks, shopping malls, or friends’ houses [21,22]. Active transportation included walking and bicycling as primary forms of PA for commuting to and from school. Mass transit and private car were not counted as active transportation The question asked was, “In what way did you usually commute to school in the last seven days?” Options were walking, by bike, by mass transit, or by private car.

#### 2.2.6. Sedentary Behavior

Sedentary behavior refers to any low-energy expenditure period while awake, such as sitting, reclining, or lying down. Increased use of motorized transportation and more screen time for work, education, and entertainment contribute to prolonged sedentary time in daily life. The question asked was, “In the past 7 days, excluding time spent in class, what was your average daily duration of sedentary activities (such as completing homework and participating in extracurricular classes, watching TV, using mobile phones, tablets, gaming consoles, computers for playing games, watching videos, or reading e-books) Options were 0 h, 0–1 h,3–5 h, 5–7 h and >7 h [18].

### 2.3. Physical Fitness

This study used the 2014 version of the Chinese National Student Physical Fitness Standards for testing. To analyze physical fitness levels, the data were divided into two groups based on the established threshold of 80 points: “excellent” (≥80 points) and “non-excellent” (<80 points). All tests were conducted by university physical education teachers who received standardized training. The test items and methods are as follows (Table 1).

This study used the 2014 version of the Chinese National Student Physical Fitness Standards (CNSPFS) [23] to assess participants’ physical fitness. Each test indicator was scored according to age- and sex-specific norms provided in the CNSPFS, with performance converted into a standardized 100-point scale. For example, sprint times, number of sit-ups, rope-skipping counts, and jump distances were converted to points according to the CNSPFS scoring tables. The overall physical fitness score was calculated as a weighted sum of the individual test indicators, with the weights varying by grade as shown in Table 1. The sum of all weighted indicators equals 100 points. Participants were then classified into two categories based on the total score: “excellent” (≥80 points) and “non-excellent” (<80 points).

All physical fitness tests were conducted by university physical education teachers who had received standardized training to ensure measurement accuracy and reliability.

### 2.4. Statistical Analysis

The data underwent double-entry verification and cleaning to ensure accuracy and validity. Data processing was performed using SPSS 27.0 (SPSS Statistics v25, IBM Corp., Armonk, NY, USA). Prior to conducting statistical analyses, the normality of continuous variables was assessed using the Shapiro–Wilk test. Descriptive statistics, including means, standard deviations, and percentages, were used to summarize basic demographic information, frequency of participation in different types of PA, and the overall rate of excellent physical fitness. A chi-square test was employed for descriptive statistical analysis, with Cramer’s V coefficient used to assess the strength of association between variables. Cramer’s V values were interpreted as follows: values less than 0.1 indicate negligible correlation, values between 0.1 and 0.3 suggest a weak correlation, values between 0.3 and 0.5 indicate moderate correlation, and values greater than 0.5 signify a strong correlation [24]. Binary logistic regression analysis was conducted using R software (version 4.4.3; R Foundation for Statistical Computing, Vienna, Austria) to assess the associations between different types and frequencies of PA and the likelihood of achieving excellent physical fitness. The dependent variable was defined as whether a student achieved excellent physical fitness or not, while the independent variables included MVPA, MSE, OSPA, OPA, active transportation, and sedentary behavior. Control variables included gender, age, school level, father’s education, mother’s education, and urban–rural classification. Cluster-robust standard errors were applied to account for potential clustering effects at the school level. A *p*-value of less than 0.01 was considered statistically significant. Before conducting logistic regression, multicollinearity was assessed using the variance inflation factor (VIF), and all variables had VIF values below 2, indicating no significant multicollinearity.

## 3. Results

A total of 102,942 participants were included in this study, with an average age of 12.9 ± 2.8 years. Among them, there were 60,555 boys, accounting for 58.8% of the total, with an average age of 13.5 ± 3.0 years, and 42,387 girls, accounting for 41.2%, with an average age of 12.0 ± 2.3 years (Table 2).

Only 3.4% of children and adolescents met the recommended 7 days/week of MVPA, while 76.7% did not engage in more than 60 min of MVPA at all. Regarding MSE, 35.7% of children and adolescents achieved the recommended frequency of ≥3 days/week, with 24.7% not engaging in MSE. Regarding organized sports, 41.1% of children and adolescents did not participate, and 23.8% engaged in such activities five or more times per week. A significant proportion (57.4%) used active transportation, with walking (44.9%) being more common than cycling (12.5%). OPA participation was relatively low, with 81.1% of children and adolescents not spending 2 h outdoors even once per week and only 8.4% reaching this time for two days per week. Additionally, sedentary behavior was prevalent, with 80.8% sitting for 0–2 h per day, followed by 11.1% sitting for 2–4 h daily.

The rate of physical fitness excellence was higher in primary school students than in middle and high school students. A Chi-square test revealed statistically significant differences in PA participation frequency based on school level, gender, and urban/rural location (see Table 3 and Table 4). For instance, organized sports frequency is weakly correlated with school level (Cramer’s V = 0.276, *p* < 0.001), while active transportation shows weak correlations with both school level (Cramer’s V = 0.209, *p* < 0.001) and gender (Cramer’s V = 0.113, *p* < 0.001). Sedentary behavior demonstrates the highest participation rate and is weakly correlated with school level (Cramer’s V = 0.129, *p* < 0.001).

**Figure 2:**
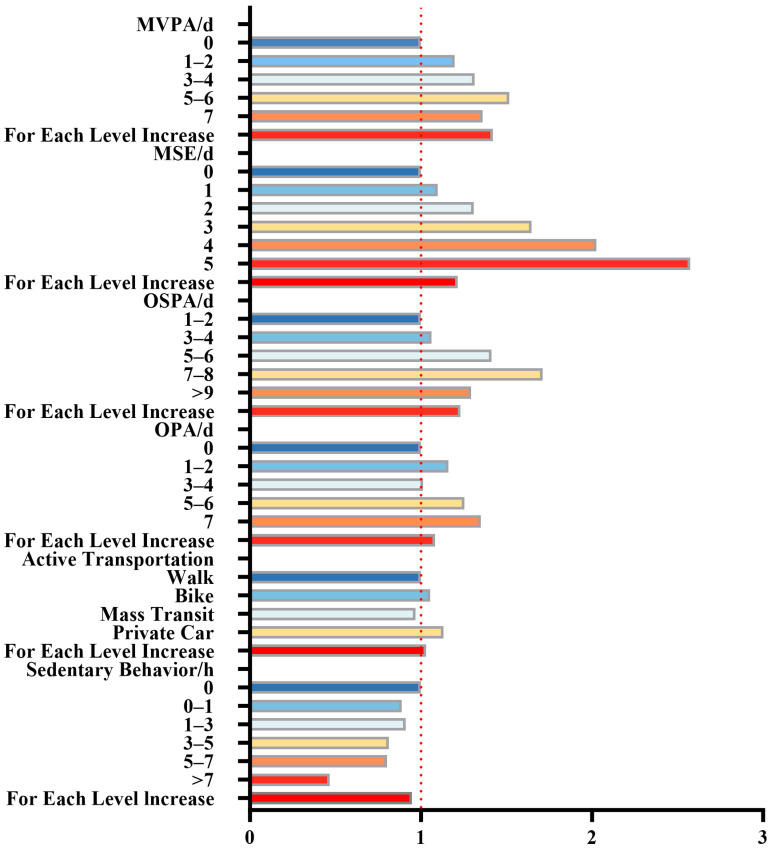
Odds Ratio of Different Types of PA Participation Frequency to Achieve Excellent Physical Fitness. Note: MVPA: moderate-to-vigorous physical activity; MSE: muscle-strengthening exercise; OSPA: organized sports and physical activity; OPA: outdoor physical activity.

The results show a significant positive correlation between the frequency of participation in MVPA and the rate of excellent physical fitness (see Figure 2 and Table 5). As the number of MVPA days increases, the probability of achieving excellent physical fitness also increases. Specifically, for each additional level of MVPA, the odds of having excellent physical fitness increase by 1.11 (95%CI: 1.08, 1.16; *p* < 0.001). Similarly, MSE frequency is also positively associated with physical fitness, with each additional day of MSE increasing the odds of achieving excellent physical fitness by 1.21 (95%CI: 1.19, 1.22; *p* < 0.001). OSPA is strongly linked to improved physical fitness, with the odds increasing by 1.23 (95%CI: 1.15, 1.32; *p* < 0.001) for each additional level of participation. For transportation, both cycling and private car use show a positive correlation with excellent physical fitness. OPA participation is positively associated, with each additional level increasing the probability by 1.08 (95%CI: 1.04, 1.13; *p* < 0.001). Lastly, sedentary behavior exhibits a negative correlation with physical fitness. For every additional level of sedentary behavior, the likelihood of achieving excellent physical fitness decreases by 0.95 (95%CI: 0.91, 0.99; *p* < 0.001).

## 4. Discussion

We observed that the participation rate in MVPA among Chinese children and adolescents is not optimistic, which is consistent with global trends [25]. The WHO recommends that children and adolescents engage in at least 60 min of MVPA daily. However, in this study, only 3.4% of the participants met this criterion. According to the Chinese PA Guidelines (2021), children and adolescents aged 6–17 should engage in MSE and bone-strengthening activities at least three days per week, but we found that only 35.7% met this requirement [26]. In comparison with global trends, the participation rates in MVPA, MSE, and OPA in our study are notably lower than those reported in countries like the United States, where higher frequencies of PA, especially in organized sports and MSE, have been shown to impact youth fitness levels positively [11]. This could be attributed to different policy frameworks, cultural contexts, and environmental factors. For instance, the Global Matrix 4.0 grades for PA in Asian countries, including China, generally report lower compliance with recommended activity levels compared to regions like Europe and North America. According to the Global Matrix 4.0, China received an overall average grade of D, lower than the Asian (C−) and global (C) averages. These results highlight critical areas that require national-level policy improvements. Compared with countries like Singapore, Japan, and South Korea, China still lags behind in several core indicators [9]. Our findings showed that primary school children demonstrated better physical fitness compared to middle and high school students [27]. This may be partly explained by their higher levels of spontaneous physical activity, lower academic burden, and more frequent participation in outdoor play and physical education classes. Additionally, younger children tend to have greater flexibility and agility relative to their body size. As children progress through the education system, increasing academic pressure and screen time may reduce physical activity levels, contributing to a decline in fitness with age [28].

The frequency of OSPA participation is relatively good, with activities organized by schools or communities, such as physical education classes, amateur sports leagues, and club sports. The Chinese Ministry of Education requires that students in grades three and above have three PE classes per week, and high school students have two per week. This may explain the relatively high compliance rate of OSPA participation in this study [29]. In terms of OPA, 81.1% of children and adolescents did not spend more than 2 h in a single session of outdoor play. According to the Global Matrix 4.0, 40–46% of children and adolescents globally meet the standard, with the lowest compliance rates in Asia. In this study, the compliance rate among Chinese children and adolescents was only 3.2%, much lower than the global average, with academic pressure cited as a key factor in Global Matrix 4.0.

In contrast, Active Transportation rates were higher and better than the international average. The global average is 40–46%, while 57.4% (44.9% walked and 12.5% cycled to school) of Chinese children and adolescents met the active transportation criteria. The Chinese Ministry of Education stipulates that students in primary and secondary schools should live within 3 km of the school, which may encourage students to choose walking or cycling as their mode of transportation [30]. Regarding sedentary behavior, Chinese children and adolescents performed relatively well, with most reporting 0–2 h of sedentary time. The excellent physical fitness rate was only 18.6%, similar to low international levels.

This study also found that MVPA, MSE, OSPA, OPA, Active Transportation, and sedentary behavior significantly impact physical fitness excellence rates. The more frequently children and adolescents engage in MVPA, OSPA, and OPA, the higher their fitness excellence rates. Regular PA is associated with better health-related physical fitness, including cardiovascular endurance, muscle strength and endurance, flexibility, and body composition, contributing to improved health outcomes [31]. Compared to MVPA and OPA, OSPA showed the strongest association with physical fitness excellence, likely because organized sports provide more structure and supervision, better guiding participants through effective, targeted exercises. OSPA often includes rigorous training programs and group collaboration, which can help improve physical fitness. The higher compliance rate of OSPA in China may explain its more significant contribution to physical fitness excellence than international standards.

Among various PA types, MSE was most strongly associated with physical fitness. In the Chinese National Student Physical Fitness Standards, activities like pull-ups, standing long jumps, sit-ups, and 50 m sprints rely heavily on MSE. Pull-ups, in particular, are a challenging test of upper-body strength with consistently low pass rates. Therefore, higher MSE frequencies were associated with better performance, thereby increasing the rate of physical fitness excellence [32]. Importantly, MSE is relevant not only to adults. In children and adolescents, regular MSE is associated with better musculoskeletal development, posture, coordination, and contributes to healthy bone growth, all of which are critical during the growth period. A higher frequency of MSE has been shown to improve performance in physical fitness tests commonly used in schools, directly supporting children’s and adolescents’ health-related physical fitness [33]. Moreover, MSE is safe and adaptable to different ages, requiring minimal equipment (e.g., bodyweight exercises, resistance bands), making it suitable for school and home settings. Importantly, MSE is not only relevant to adults. In children and adolescents, regular MSE supports musculoskeletal development, improves posture, enhances coordination, and contributes to healthy bone growth, all of which are critical during growth. Higher frequency of MSE has been shown to improve performance in physical fitness tests commonly used in schools, directly supporting children’s and adolescents’ health-related physical fitness. Moreover, MSE is safe and adaptable to different ages, requiring minimal equipment (e.g., bodyweight exercises, resistance bands), making it suitable for school and home settings, further highlighting MSE’s unique long-term health benefits. Self-reported MSE is also more reliable than MVPA, which makes it particularly important to prioritize MSE for children and adolescents [34]. The frequency and intensity of muscle training are relatively straightforward to measure, whereas aerobic exercise may be influenced by more external variables (such as environment and equipment). Thus, prioritizing MSE in physical fitness evaluations is especially important for improving the physical fitness of children and adolescents.

While MSE and MVPA offer significant health benefits, research shows that combining the two is linked to better outcomes. People who engage in both MSE and MVPA experience better outcomes in reducing chronic disease incidence, improving fitness levels, and enhancing mental health than those who only engage in MVPA [35]. Active Transportation, such as walking and cycling, also positively affects physical fitness. Children and adolescents who walk or cycle tend to have better cardiovascular and muscular endurance than those who use passive transportation modes [36,37]. However, this study also found that using private cars was positively correlated with physical fitness excellence, even more so than cycling. It is possible that this relationship is influenced by unmeasured confounding factors such as family socioeconomic status, access to organized sports, or parental support [38]. Additionally, students who use private cars may participate in other forms of PA outside of school, compensating for their lack of active commuting [39]. We acknowledge that our study did not conduct a stratified analysis of OSPA levels among children and adolescents who used private cars versus those who engaged in active transportation. This limits our ability to fully explain the observed association between private car use and physical fitness. Future research should explore the interaction between transportation modes and participation in organized physical activities to better understand these patterns.

Many studies have confirmed the close association between increased sedentary time and declining health [40,41]. Sedentary behavior is negatively associated with physical fitness, particularly cardiovascular health [42], obesity [43,44], and bone health [45]. Reducing sedentary time and increasing PA frequency are considered critical measures for promoting the health of children and adolescents [46]. In this study, longer sedentary time was associated with lower physical fitness excellence rates, consistent with previous research [47]. Sedentary behavior includes watching TV, playing on computers, using smartphones, and playing video games, all closely related to declining fitness levels, especially in obesity, metabolic health [48], and cardiovascular health [49].

The Global Matrix 4.0 provides an international benchmark for assessing PA in children and adolescents. Comparing our findings to these benchmarks, Chinese children and adolescents exhibit relatively high participation in OSPA and active transportation, but very low engagement in outdoor play, with only 3.2% meeting recommended durations. These results are consistent with trends observed in other Asian countries, where academic pressure and urbanization constrain opportunities for outdoor activities. By situating our study within the Global Matrix framework, we can identify specific areas where Chinese youth fall behind global standards, highlighting priorities for policy interventions and targeted promotion of PA [10,17].

In summary, this study confirms the multifaceted impact of different types of PA on physical fitness, emphasizing the necessity of diverse activity forms to improve the physical fitness of children and adolescents. Therefore, reducing sedentary time, encouraging outdoor activity, limiting daily screen time to less than one hour (as recommended by the World Health Organization and other health guidelines for children and adolescents), and creating more opportunities for outdoor play, particularly by fostering easy access to sports in schools and families, are essential. Strengthening organized sports activities, such as those provided by schools and communities through PE classes, sports clubs, and extracurricular sports training, will help children and adolescents develop regular exercise habits. Additionally, encouraging walking or cycling to school will increase daily PA levels. To improve adolescent physical fitness, it is essential that national health policies, such as “Healthy China 2030”, incorporate strategies that increase access to organized sports and PA opportunities, especially in schools and communities.

This study presents several strengths. (1) Large Sample Size: This study included a substantial sample size of over 100,000 children and adolescents aged 9–18, which provides a robust dataset for drawing statistically significant conclusions. This large sample increases the power of the findings and their generalizability to broader populations. (2) Comprehensive Analysis of PA Types: By examining a variety of physical activities, including MVPA, MSE, OSPA, OPA, and Active Transportation, the study offers a detailed view of the multiple facets of PA that contribute to physical fitness among children and adolescents.

This study has several limitations that should be acknowledged. First, as a cross-sectional study, only associations between different types of PA—such as MVPA, MSE, and OSPA—and physical fitness can be established. Causal relationships cannot be inferred, and reverse causality is possible. For instance, adolescents with higher levels of physical fitness may be more likely to participate in physical activities, especially those that require higher physical competence, such as organized sports or MSE. Longitudinal studies are needed to determine these relationships’ direction and magnitude better. Second, the majority of PA data were self-reported, which introduces potential biases, including recall bias and social desirability bias, possibly leading to over- or underestimation of actual activity levels. These measurement errors should be considered when interpreting the results. In future research, objective measures, such as accelerometers, are recommended to improve data accuracy and reduce subjectivity. Third, the questionnaire did not capture certain types of daily PA—such as leisure shopping, household chores, running errands, and volunteering. These unmeasured activities may contribute to overall PA levels and influence physical fitness outcomes. Fourth, while some socioeconomic factors such as parental education and urban–rural classification were included as control variables, the study did not systematically examine regional or socioeconomic disparities in PA and fitness outcomes. Future studies should explore how contextual factors like income level, geographic setting, or school infrastructure affect PA engagement. Fifth, this study did not assess other significant covariates such as nutrition, sleep patterns, and family support for PA. However, these factors are known to influence physical fitness and health outcomes. Lastly, 24,647 participants were excluded due to missing or invalid data across key variables, such as PA indicators and fitness tests. These missing data were not completely random and were largely due to structured programmatic differences, including those related to boarding school systems. As a result, multiple imputation was not applied. This selective exclusion may introduce bias and should be considered when generalizing the findings to the broader population. Although this study did not focus on subgroup differences, future studies should investigate how age and gender may moderate the relationship between PA and fitness, as tailored interventions may be more effective for different population groups.

## 5. Conclusions

This study demonstrates that physical activities such as MVPA, MSE, OSPA, OPA, and Active Transportation are significantly associated with higher physical fitness of children and adolescents. Our findings indicate that OSPA and MSE showed the strongest associations with better fitness outcomes. Conversely, sedentary behavior was associated with lower physical fitness rates. Therefore, based on our findings, it would be beneficial to encourage participation in various physical activities, particularly OSPA and MSE, while also considering strategies to reduce sedentary time to improve physical fitness in this population.

## Figures and Tables

**Table 1:** Physical Fitness Test Indicators for Different Grades.

Grades	Test Indicators	Weighting (%)
Grades from Primary to High School	BMI	15
	Vital capacity	15
	50 m sprint	20
Grade 4 of Primary School	Sit and reach	20
	One minute of rope-skipping.	20
	One minute of sit-ups	10
Grades 5 and 6 of Primary School	Sit and reach	10
	One minute of rope-skipping.	10
	One minute of sit-ups	20
	50 m × 8 shuttles run	10
Grades from Middle School to High School	Sit and reach	10
	Standing long jump	10
	Pull-ups(boys)/One minute of sit-ups(girls)	10
	1000 m and 800 m run	20

Note: The sum of all weighted indicators equals 100 points. Body Mass Index (BMI) = weight (kg)/height^2^ (m^2^).

**Table 2:** Basic Information of Participants.

	Total n (%)	Age/y Mean ± SD	Primary School n (%)	Middle School n (%)	High School n (%)	Urban n (%)	Rural n (%)
Boys	60,555 (58.8)	13.5 (3.0)	20,496 (47.9)	19,399 (57.2)	20,660 (78.6)	31,511 (60.2)	29,044 (57.4)
Girls	42,387 (41.2)	12.0 (2.3)	22,276 (52.1)	14,496 (42.8)	5615 (21.4)	20,849 (49.8)	21,538 (42,6)
Total	102,942	12.9 (2.8)	42,772 (41.6)	33,895 (32.9)	26,275 (25.5)	52,360 (50.9)	50,582 (49.1)

Note: y: years; SD: standard deviation.

**Table 3:** Survey of Different Types of PA Participation Frequency and Excellence Rate of Physical Fitness.

	Total (%)	Primary School (%)	Middle School (%)	High School (%)	Boys (%)	Girls (%)	Urban (%)	Rural (%)
MVPA/d		Cramer’s V = 0.069 **			Cramer’s V = 0.051 **		Cramer’s V = 0.017 **	
0	76.7	72.4	79.7	80.0	74.9	79.3	76.1	77.4
1–2	12.7	15.5	10.8	11.0	13.7	11.4	13.2	12.4
3–4	3.1	3.6	2.8	2.3	3.2	2.7	3.1	3.1
5–6	4.1	4.0	4.2	4.2	4.5	3.5	4.2	4.1
7	3.4	4.5	2.5	2.6	3.7	2.9	3.6	3.2
MSE/d		Cramer’s V = 0.098 **			Cramer’s V = 0.062 **		Cramer’s V = 0.059 **	
0	24.7	21.7	21.9	33.2	24.5	25	23.5	26
1	18.2	17.7	18.3	19.0	17.3	19.5	17.5	25.7
2	21.3	20.9	21.9	21.3	20.7	22.2	20.9	21.8
3	15.5	16.5	17.5	11.2	15.3	15.7	15.8	15.2
4	6.7	7.9	6.8	4.6	6.9	6.4	7.4	6
>5	13.5	15.3	13.6	10.7	15.2	11.2	15	12
OSPA/d		Cramer’s V = 0.276 **			Cramer’s V = 0.094 **		Cramer’s V = 0.057 **	
0	4.3	3.6	3.2	6.9	5.0	3.4	3.5	5.2
1–2	36.5	21.0	34.9	63.9	39.8	32.0	35.0	38.0
3–4	45.9	54.8	51.2	24.3	43.2	49.7	48.0	43.6
5–6	12.6	19.3	10.2	4.6	11.5	14.1	12.6	12.4
7	0.8	1.3	0.5	0.3	0.7	0.9	0.8	0.7
OPA/d		Cramer’s V = 0.044 **			Cramer’s V = 0.039 **		Cramer’s V = 0.033 **	
0	81.1	78.7	81.7	84.0	79.8	82.8	80.1	82.0
1–2	11.4	12.3	11.4	10.0	12.3	10.1	11.6	11.2
3–4	0.9	0.9	0.9	0.8	0.9	0.7	0.9	0.9
5–6	3.4	3.9	3.1	3.1	3.6	3.3	3.8	3.1
>7	3.2	4.2	2.9	2.2	3.3	3.1	3.7	2.8

NOTE: MVPA: moderate-to-vigorous physical activity; MSE: muscle-strengthening exercise; OSPA: organized sports and physical activity; OPA: outdoor physical activity; **: *p* < 0.01.

**Table 4:** Survey of Different Types of PA Participation Frequency and Excellence Rate of Physical Fitness, continued.

	Total (%)	Primary School (%)	Middle School (%)	High School (%)	Boys (%)	Girls (%)	Urban (%)	Rural (%)
Active transportation		Cramer’s V = 0.209 **			Cramer’s V = 0.113 **		Cramer’s V = 0.063 **	
Walk	44.9	53.8	41.1	35.2	Walk	44.9	53.8	41.1
Bike	12.5	4.5	17.6	18.8	Bike	12.5	4.5	17.6
Mass Transit	19.5	12	21.6	29	Mass Transit	19.5	12	21.6
Private Car	23.1	29.6	19.7	17	Private Car	23.1	29.6	19.7
Sedentary behavior/h		Cramer’s V = 0.055 **			Cramer’s V = 0.032 **		Cramer’s V = 0.050 **	
0	4.7	5.3	4.5	4.	4.8	4.5	4.5	4.9
0–1	80.8	82.6	79.7	79.2	79.8	82.1	81.7	79.8
1–3	11.1	8.8	12.7	12.7	11.6	10.3	11.2	11.0
3–5	1.1	1.2	1.3	0.8	1.2	1.	0.9	1.3
5–7	0.2	0.3	0.2	0.2	0.2	0.2	0.2	0.2
>7	2.1	1.9	1.7	3.1	2.4	1.8	1.5	2.8
Excellence rate		Cramer’s V = 0.129 **			Cramer’s V = 0.007 **		Cramer’s V = 0.004 **	
Excellent	18.6	23.9	17.4	11.4	18.3	18.9	18.7	18.4
Non-excellent	81.4	76.1	82.6	88.6	81.7	81.1	81.3	81.6

NOTE: **: *p* < 0.01.

**Table 5:** ORs (95% CI) of physical fitness excellence by PA type and frequency.

	OR	CI_Low	CI_High
MVPA/d			
0	1.00	1.00	1.00
1–2	1.20	1.09	1.31
3–4	1.31	1.11	1.56
5–6	1.52	1.34	1.71
7	1.36	1.13	1.64
For Each Level Increase	1.11	1.08	1.16
MSE/d			
0	1.00	1.00	1.00
1	1.10	1.01	1.19
2	1.31	1.20	1.42
3	1.65	1.49	1.81
4	2.03	1.82	2.25
>5	2.57	2.29	2.89
For Each Level Increase	1.21	1.19	1.24
OSPA/d			
0	1.00	1.00	1.00
1–2	1.06	0.88	1.28
3–4	1.41	1.16	1.72
5–6	1.71	1.34	2.18
7	1.29	0.96	1.75
For Each Level Increase	1.23	1.15	1.32
OPA/d			
0	1.00	1.00	1.00
1–2	1.16	1.07	1.26
3–4	1.01	0.77	1.33
5–6	1.25	1.06	1.49
7	1.35	1.12	1.62
For Each Level Increase	1.08	1.04	1.13
Active Transportation			
Walk	1.00	1.00	1.00
Bike	1.05	0.95	1.17
Mass Transit	0.97	0.89	1.06
Private Car	1.13	1.03	1.25
For Each Level Increase	1.03	1.00	1.06
Sedentary Behavior/h			
0	1.00	1.00	1.00
0–1	0.89	0.74	1.07
1–3	0.91	0.75	1.10
3–5	0.81	0.67	0.99
5–7	0.80	0.61	1.05
>7	0.47	0.18	1.20
For Each Level Increase	0.95	0.91	0.99

NOTE: MVPA: moderate-to-vigorous physical activity; MSE: muscle-strengthening exercise; OSPA: organized sports and physical activity; OPA: outdoor physical activity.

## Data Availability

The datasets generated and/or analyzed during the current study are not publicly available due to confidentiality of subject information but are available from the corresponding author on reasonable request.

## Abbreviations

The following abbreviations are used in this manuscript:

PA	Physical activity
MVPA	Moderate-to-vigorous physical activity
OSPA	Organized sports and physical activity
OPA	Outdoor physical activity
MSEs	Muscle-strengthening exercises
BMI	Body Mass Index
VIF	Variance inflation factor
